# Oral microbial changes, oral mucositis and febrile neutropenia during myelosuppressive chemotherapy in patients diagnosed with a solid tumor or lymphoma

**DOI:** 10.3389/froh.2024.1461463

**Published:** 2024-11-14

**Authors:** Judith A. E. M. Zecha, Judith E. Raber-Durlacher, Bernd W. Brandt, Mark J. Buijs, Egija Zaura, Jan de Lange, Ludwig E. Smeele, Alexa M. G. A. Laheij

**Affiliations:** ^1^ Department of Oral and Maxillofacial Surgery, Amsterdam UMC, University of Amsterdam, Amsterdam, Netherlands; ^2^ Department of Oral Medicine, Academic Center for Dentistry Amsterdam, University of Amsterdam and Vrije Universiteit Amsterdam, Amsterdam, Netherlands; ^3^ Department of Preventive Dentistry, Academic Center for Dentistry Amsterdam, University of Amsterdam and Vrije Universiteit Amsterdam, Amsterdam, Netherlands; ^4^ Department of Head and Neck Oncology and Surgery, Netherlands Cancer Institute—Antoni van Leeuwenhoek, Amsterdam, Netherlands

**Keywords:** oral microbiota, febrile neutropenia, dental infection, oral mucositis, myelosuppressive chemotherapy, solid tumor

## Abstract

**Objectives:**

To evaluate the possible changes of the oral microbiome during myelosuppressive chemotherapy (CT) and to investigate the potential relationship between the oral microbiome, the presence of oral mucositis (OM) and febrile neutropenia (FN).

**Methods:**

A prospective, longitudinal, observational study was conducted in patients receiving myelosuppressive CT for a solid tumor or lymphoma. Oral rinsing samples were retrieved before, during and after the start of CT, but also when OM or FN was present. The samples were analyzed using 16S rRNA gene amplicon sequencing and statistical analysis was performed using alpha (Shannon) and beta (PERMANOVA) diversity analyses. Furthermore, differential abundances were analyzed using ALDEx2v1.32.0. Differences between groups were calculated using the Mann Whitney *U*-test, Kruskal-Wallis test and Wilcoxon Signed Rank using R.

**Results:**

Forty-six patients, with a mean follow up of 114 days, were included for analysis and a total of 138 oral rinsing samples were available in the CLR-transformed data for PERMANOVA and 137 samples—for alpha diversity calculation. Significant changes in alpha diversity were seen when OM or FN was present. Moreover, significant changes were seen in beta diversity during the course of the CT treatment and when OM was present. Genera showing substantial changes in relative abundance were *Streptococcus* during the course of CT treatment and *Prevotella, Fusobacterium, Selenomonas, Actinomyces* and *Leptotrichia* when OM was present.

**Conclusion:**

Changes in the oral microbiome were observed during the CT-regimen and when OM was present. Furthermore, changes of the oral microbiota during FN episodes were observed; however, larger studies should be performed to substantiate our results.

## Introduction

The role of the (oral) microbiome in health and disease is becoming evident (1, 2). Oral homeostasis is maintained due to complex interactions between the tissues of the host and resident microorganisms (3). In the oral cavity, about 700 bacterial taxa can be identified and each individual harbors about 100 different taxa known as commensal or resident oral bacteria (2–4). When the equilibrium, in which resident species in the oral cavity maintain a healthy state, becomes disturbed a dysbiotic shift occurs. This results in a disbalance and domination of a few species, that can grow to higher proportions than under healthy conditions, associated with an higher risk of disease (3). A disturbance of homeostasis can occur, for instance, due to salivary gland dysfunction, poor oral hygiene, a change in diet or smoking habits, oral or systemic diseases or medications (2, 5).

In cancer patients, a possible cause of a dysbiotic shift of the oral microbiome includes cytotoxic therapy such as chemotherapy (CT) and curative radiotherapy to the head and neck.

Myelosuppressive CT is used in the majority of cases for the treatment of solid tumors or lymphoma (6). This treatment modality has many systemic side effects, particularly affecting tissues with a high mitotic activity (6). One of the side effects of myelosuppressive CT is severe neutropenia, during which patients are unable to mount an effective inflammatory response. This poses these patients at risk of developing fever which may herald a potentially dangerous infection.

Febrile neutropenia (FN) is defined as fever during neutropenia during the course of CT which can lead to unplanned hospital admissions, and is associated with higher morbidity, delay or cancellation of the CT and even death (7). While an infection is the most probable cause of this severe side effect, causative pathogens are not identified in many cases (8). Common sites of infection are the skin, lungs and/or urine tract (9), but the oral cavity may also play a role (8). To date, little is known about the composition of the oral microbiome during episodes of FN.

Another common adverse effect of CT is oral mucositis (OM) (10). Oral mucositis is an inflammatory condition with damage to the oral mucosal lining due to direct and indirect effects of CT and/or radiotherapy (11). Clinically, CT-induced OM presents as redness and/or ulcerations of the non-keratinized oral mucosa. OM can lead to pain, reduced oral intake, a lower quality of life and delay or cancellation of CT (10, 12, 13).

The development of OM is complex and can be divided in five interrelated stages: initiation phase, primary damage response, signal amplification, ulceration and, finally, the healing phase (14). The initiation phase consists of direct physical DNA damage and triggering of biological events due to CT or radiotherapy. This results in activation of the innate immune response and apoptosis. Next, transcription factors including Nuclear Factor kappaB are activated leading to the generation of proinflammatory cytokines contributing to ulceration. These ulcerations may be colonized by mainly Gram-negative bacteria, which may in turn contribute to the production of more proinflammatory cytokines thereby aggravating inflammation. In most cases, the healing phase occurs spontaneously approximately after 2–4 weeks after the start of a CT cycle and generally simultaneously with neutrophil recovery.

Studies suggest potential associations between changes of the oral microbiota and development of OM and bloodstream infections as bacteria may translocate into the circulation via ulcerated tissue, particularly in myelosuppressed patients (6, 8, 12, 13, 15–19).

With respect to this, it has been reported that mucosal injury is associated with a reduction in abundance of commensal bacteria occurs and a potential enrichment of pathogenic microorganisms with virulence factors like lipopolysaccharide, fimbriae and proteolytic metabolites (20). Laheij et al. (21) found a lower alpha diversity, known as the microbial diversity within a single sample (22), and dysbiosis of the oral microbiota associated with OM in patients undergoing hematopoietic cell transplantation, and recovery of the microbiota after completion of the treatment (23).

In addition to an infectious cause of FN, it has been suggested that the inflammation associated with OM (and mucositis of the rest of the gastrointestinal tract) is also a significant cause of fever induced by inflammatory products entering the circulation (24, 25).

Another route by which oral microorganisms may contribute to FN, is via pre-existing oral inflammatory diseases, such as marginal or apical periodontitis. Microorganisms, bacterial cell wall substances and inflammatory products located in ulcerated periodontal pockets or peri-apically can cause local infectious exacerbations and may enter the circulation and spread to other body parts which may eventually lead to systemic inflammation and fever (26). In myelosuppressed cancer patients, pre-existing oral pathologies have been suggested to cause life-threatening infectious complications (27, 28).

Currently, only one study reports on oral microbiome changes in patients receiving myelosuppressive CT for solid tumors (29). This longitudinal, prospective study in breast cancer patients reports no statistical difference in alpha and beta diversity indices before, during and after CT. Beta diversity describes the measure of the (dis)similarity of two communities, thus a between-sample diversity (22). The prevalence of OM and FN and their possible association with oral microbial changes has not been part of this study.

As very little is known about the relationship between the oral microbiome, FN and OM, the aim of this study was to evaluate the oral microbial changes during myelosuppressive CT and possible associations with OM and FN in patients diagnosed with a solid tumor or lymphoma.

## Material and methods

This prospective longitudinal observational study was performed at the Department of Oral and Maxillofacial Surgery and the Department of Oncology of the Amsterdam University Medical Center, location AMC. The Institutional Review Board approved this study (NL53440.018.15). All participants signed a written informed consent. This study is part of a larger prospective longitudinal observational study (9, 30). The clinical results of the potential role of dental foci and OM in relation to development of FN is reported by Zecha et al. (9) and the additional diagnostic value of the panoramic radiograph in pre-chemotherapy dental screening is reported by Zecha et al. (30).

### Patient inclusion

Patients older than 18 years, with a (partial) natural dentition and/or dental implants, no prior head and neck radiotherapy, diagnosed with a solid tumor outside of the head and neck region or lymphoma and scheduled for CT-treatment with an intermediate risk of FN (31) were eligible for inclusion.

### Study flow and retrieval of the oral rinsing samples

After a written consent, patients underwent an oral examination including orthopantomogram, in which pre-existing dental and oral pathology was recorded. No other x-rays were taken. Specification of the oral examination and oral focus definitions are described elsewhere (9, 30). The patient demographics—gender, age, intoxications, medication use, American Society of Anesthesiologists (ASA) classification, World Health Organization (WHO) performance status and cancer diagnosis—were retrieved from the patient records.

During the pre-treatment dental evaluation, a baseline rinsing sample was retrieved. During the courses of the CT-regimen, multiple rinsing samples were retrieved at the same time as when the oral cavity was scored for the presence of OM, according to the CTC-AEv3.0 guidelines (32). Collection of the oral rinsing samples took place just before CT-infusion and during regular check-ups with the oncologist. The examiners (one dentist, three dental students, one resident of oral maxillofacial surgery and one oral hygienist) were trained in reliable and consistent OM scoring and (also) received an instruction card. When the CT-regimen was completed and patients visited the oncologist for their regular check-ups, a final oral rinsing sample was retrieved.

If a patient presented with fever to the emergency care, OM was also assessed and an oral rinsing sample was taken by one of the examiners. In addition, patients were examined intraorally for oral fungal and recrudescent herpes simplex virus (re)infection and acute exacerbations of dental infections.

### Chemotherapy regimens

Chemotherapy regimens and the number of planned CT cycles were recorded. Changes in treatment plan were also registered. Dose delay was defined as a delay of planned chemotherapy for more than 3 days. Dose reduction was defined as an administered dose that was 85% or less of the initially planned dose (33). A chemotherapy cancellation was defined as an initially planned dose that was not given at all.

Despite the strict inclusion criteria for FN risk (31), the actual risk of neutropenia varied between CT regimens. For analysis we therefore divided the group, before analysis, in relatively low- and relatively high risk of myelotoxicity based on the administered CT agent given [see Appendix 1 in Zecha et al. (9).]. This classification was performed by an experienced oncologist.

### Pre-chemotherapy oral screening

Prior to the start of CT, an oral examination took place consisting of the following:

• Evaluation of dental mindedness (dental visits, oral hygiene habits) and oral complaints over the last 3 months
• Intra-oral screening for dental and/or mucosal pathology
• Periodontal screening using the Dutch Periodontal Screening Index (34)
• Screening for peri-implant mucositis and peri-implantitis
• Panoramic radiograph for all patients followed by selective peri-apical radiographs if indicated

Pre-existing dental and oral pathologies that may contribute to the development of FN and infectious complications, were noted as an oral focus in accordance with the guidelines of the Dutch Association of Maxillofacial Surgery (35). These included:

• Periodontal disease (DPSI 4; periodontal probing depth of ≥6 mm; peri-implantitis was also considered as a focus)
• Profound dental caries; caries reaching far into the dentin
• Periapical pathology
• (Partially) impacted teeth
• Retained roots with surrounding pathology

Treatment of foci was only considered at the discretion of the dentist and/or when patient reported pain or any other symptoms.

### OM and timing of sample collection

OM was scored according to the CTC-AEv3.0 (32), during the CT regimen and after CT was completed. OM was graded as 1 (erythema), 2 (patchy ulcerations or pseudomembranes), 3 (confluent ulcerations or pseudomembranes, bleeding with minor trauma), 4 (tissue necrosis, significant bleeding, life threatening consequences) or 5 (death). All examiners were trained in reliable and consistent OM scoring and received an instruction card. Oral rinsing samples were taken during the period that OM was present. When a patient presented with fever, OM was also assessed and an oral rinsing sample was retrieved.

### Febrile neutropenia

Febrile neutropenia was defined as temperature ≥38.5°C or two consecutive readings of >38.0°C for 2 h and an absolute neutrophil count <500/*μ*l or expected to fall below this threshold (36, 37). When FN was diagnosed, laboratory and/or radiological results (including full hematological blood count, infection panel, urine sediment and chest x-ray), working diagnosis and treatment plan, including the antibiotic regimen, were noted. The set of blood cultures, collected during the fever episode and placed in an incubator for aerobic and anaerobic growth, were examined for the presence of microbial growth after 2 days. Sepsis/septic shock and/or death was also noted.

### Collection of samples

An oral rinsing sample was taken by using a sterile tube of 10 ml with 0.9% sterile saline solution. The patient was asked to rinse the oral cavity for 30 s and to spit the rinse in a sterile container. These containers were pelleted by centrifugation immediately after collection (7 min at 4,500 × g), and resuspended in sterile 1 ml PBS and stored at −80°C until analysis.

### Sample processing and 16s rRNA gene amplicon sequencing

The tubes with oral rinsing samples were thawed and centrifuged. The pellets were resuspended in 100 *µ*l TE buffer (Tris-EDTA) and transferred to a 96-well deepwell plate. After addition of 100 *µ*l Lysis buffer (LGC genomics GmbH, Berlin, Germany), 250 *µ*l 0.1 mm Zirconia beads (Biospec, Bartlesville, OK, USA), and 200 *µ*l RotiPhenol (Carl Roth, Karlsruhe, Germany), the mixture was subjected to four bead-beating steps of 2 min each. DNA extraction and purification was performed with the LGC Magmini kit (LGC genomics GmbH), after which the bacterial DNA concentration was determined by a 16S ribosomal RNA gene quantitative polymerase chain reaction (qPCR) (38). For each sample, 1 ng of DNA was amplified, with barcoded forward and reverse primers (39), using the 16S rRNA gene-specific sequences V4F/515F: GTGCCAGCMGCCGCGGTAA and V4R/806R: GGACTACHVGGGTWTCTAAT (40). Paired-end sequencing (2 × 251 nt) was conducted on the Illumina MiSeq platform with the MiSeq Reagent Kit v3 at the Core Facility Genomics, Amsterdam UMC. The flow cell was loaded with 8 pmol, including 30% PhiX.

The reads were quality-filtered, denoised, mapped to zero-radius operational taxonomic units (zOTUs), and assigned taxonomy using the HOMD database (v14.51) (41), as described earlier (42). Control samples consisted of PBS solution (used for storage of the samples), sterile 0.9% NaCl solution (used to rinse with), blank DNA isolations, and PCR controls (controls are used to detect possible contamination of all carriers used in the sequencing process).

### Statistical analysis

For the calculation of the alpha diversity, the final zOTU table was randomly subsampled at 9,500 reads/sample. One sample was lost due to too few reads after subsampling at 9,500 reads/sample. For PERMANOVA, the zOTU-table was centered-log ratio (CLR) transformed, using a pseudocount of 0.5 and a minimal sample depth of 2,600 reads.

Permutational multivariate analysis of variance (PERMANOVA; Aitchison distance as Euclidean distance on the CLR-transformed data, 99,999 permutations), and PERMANOVA with permutations restricted to the subject were performed using R v4.3.1 (43) and the R packages vegan v2.6–4 (44), microbiome v1.22.0 (45) and phyloseq v1.44.0 (46). The (unbiased) Shannon diversity index was calculated using PAST (47). Differential abundance analyses were carried out with the non-subsampled data, as for the CLR transformation above, using ALDEx2v1.32.0 (48, 49).

Differences between groups were calculated using the Mann Whitney *U*-test and Kruskal-Wallis test for unrelated samples (followed by Dunn's *post-hoc* test) and Wilcoxon Signed Rank test for related samples using *R*. The relations between independent variables and the longitudinally measured Shannon diversity index of the oral microbiota were analyzed using Linear Mixed Model Analysis for continuous outcome values, to correct for the dependency in outcome measurement. Independent variables were entered as fixed effects. SPSS version 26 (IBM) was used for these analyses. A *p*-value < 0.05 was considered statistically significant.

## Results

**Figure 1:**
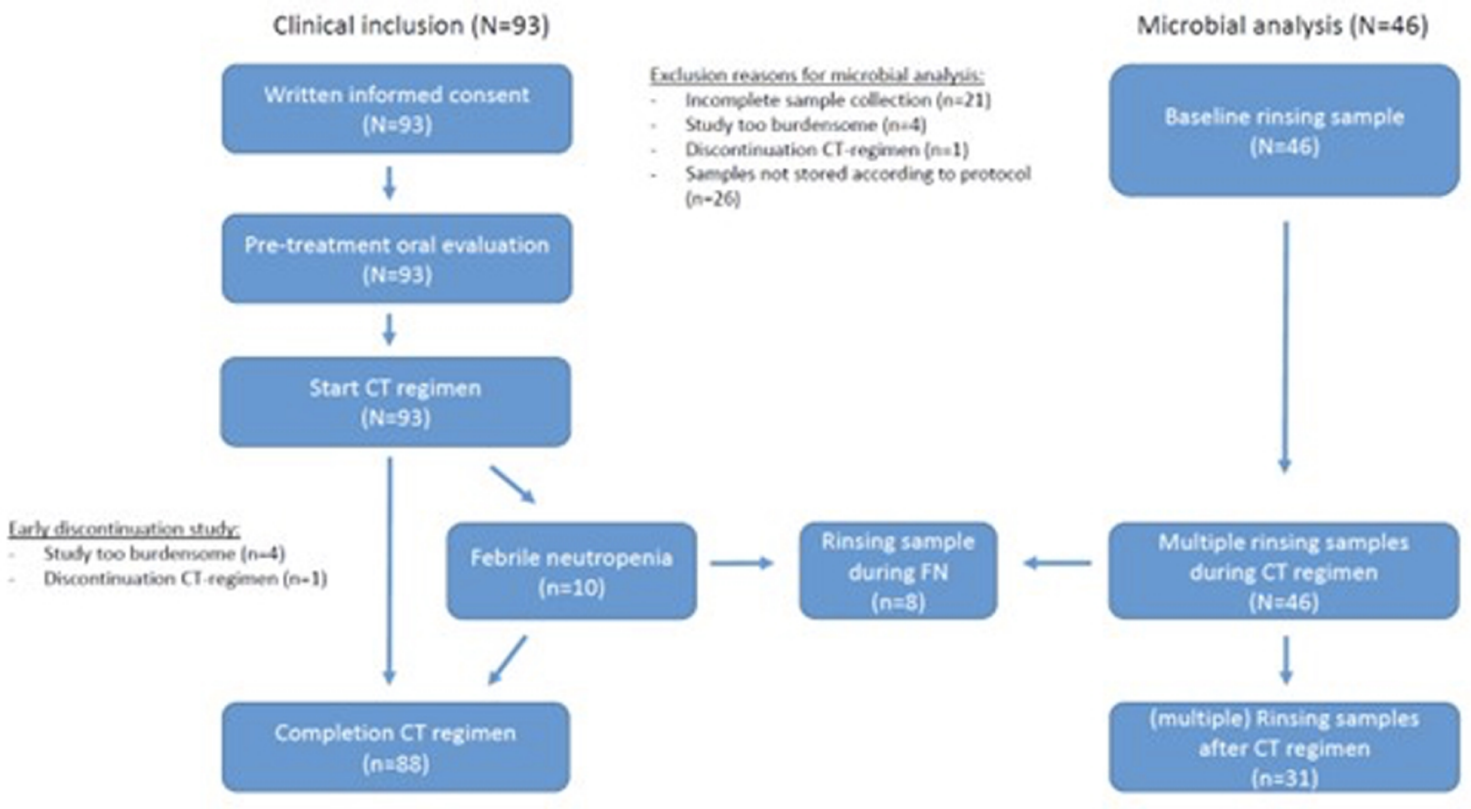
Flowchart of the study, showing the study design.

In this study, 93 patients were eligible for inclusion. However, for microbial analysis twenty-one patients could not be included due to several reasons: incomplete sample collection (*n* = 16), continuing with the study was too burdensome (*n* = 4) and early discontinuation of the treatment (*n* = 1). In 26 patients, the samples were not stored according to the protocol and therefore were excluded from analysis (Figure 1). Finally, samples of 46 patients, all with a baseline sample, were available for oral microbiota analysis with a total of 138 oral rinsing samples available in the CLR-transformed data for PERMANOVA and 137 samples—for alpha diversity calculation.

### Patient characteristics

In Table 1 patient demographics and tumor/treatment characteristics are described. The majority of the included patients were female and most patients were diagnosed with a gynecological tumor, followed by a tumor in the upper GI tract. Details about the administered CT regimens is reported in Appendix 1. Alterations of the treatment plan were performed in 19 patients and dose reductions were necessary in 9 patients. The mean follow-up period was 114 days (range 28–200).

**Table 1:** Patient demographics; and tumor and treatment characteristics (*N* = 46).

		No. of patients (*N*)	Percentage (%)
Gender	Male	16	34.8
	Female	30	65.2
Age	Mean 51.6 years, range 18–78 years, SD 16.1		
	18–40	13	
	41–59	15	
	60–80	18	
Smoking	Yes	6	13.0
	No	27	58.7
	Quit	13	28.3
Alcohol use	Yes	8	44.4
	No	10	55.6
Tumor subgroup	Gynecological	20	43.5
	Upper GI tract	9	19.6
	Sarcoma	7	15.2
	Lymphoma	4	8.7
	Urinary tract	3	6.5
	Breast	2	4.3
	Lower GI tract	1	2.2
CT-regimen	Relatively high risk	21	45.7
	Relatively low risk	25	54.3
Prophylactic G-CSF	Yes	7	15.2
	No	39	84.8
Dose reduction	Yes	9	19.6
	No	37	80.4
CT cycles alterations	Delay	11	23.9
	Cancellation	8	17.4
	No alterations	27	58.7

CT, chemotherapy; G-CSF, granulocyte colony-stimulating factor.

### Clinical outcomes

#### Dental/oral pathologies

During the pre-treatment oral evaluation, a dental focus was found in 22 patients (47.8%) of which 18 patients had a periodontal focus. None of these asymptomatic dental foci were treated prior to the start of the CT regimen. In 15 patients, peak OM was grade 2 (ulcerations) and in 14 patients peak OM grade was 1 (erythema), at any time during any CT cycle. No OM was present at the evaluation when the CT regimen was completed, see Table 2. The results of the pre-treatment oral evaluation of the full cohort is published elsewhere (9).

**Table 2:** Oral pathologies; dental focus before start CT regimen and OM score during CT regimen.

Oral pathologies (*N* = 46)			
		No. of patients (*N*)	Percentage (%)
Dental focus	Yes	22	47.8
	Periodontitis (pocket ≥6 mm)	18	
	Periapical	13	
	Profound caries	4	
	Root remnant	4	
	Partial impacted tooth	2	
	No	24	
			52.2
Highest reported mucositis score	Grade 1	14	30.4
	Grade 2	15	32.6
	No mucositis	17	37.0

#### Neutropenic fever

Six patients developed fever (≥38.5°C) while neutropenic (M:F; 2:4, age 18–78). All these patients received a CT regimen with a relatively higher risk of developing neutropenia. The mean neutrophil count was 0.09 × 10^9^/L (0.00–0.33). For one patient, the CT course was cancelled and for two it was delayed. Three patients who developed fever (50%), received CT for an osteosarcoma. Of the latter patients, a dental focus was found during the dental examination in two patients (33%), four patients reported salivary changes (67%) and all patients presenting with FN had developed oral mucositis during the CT regimen, of which five patients grade 2.

### Microbial analysis

**Figure 2:**
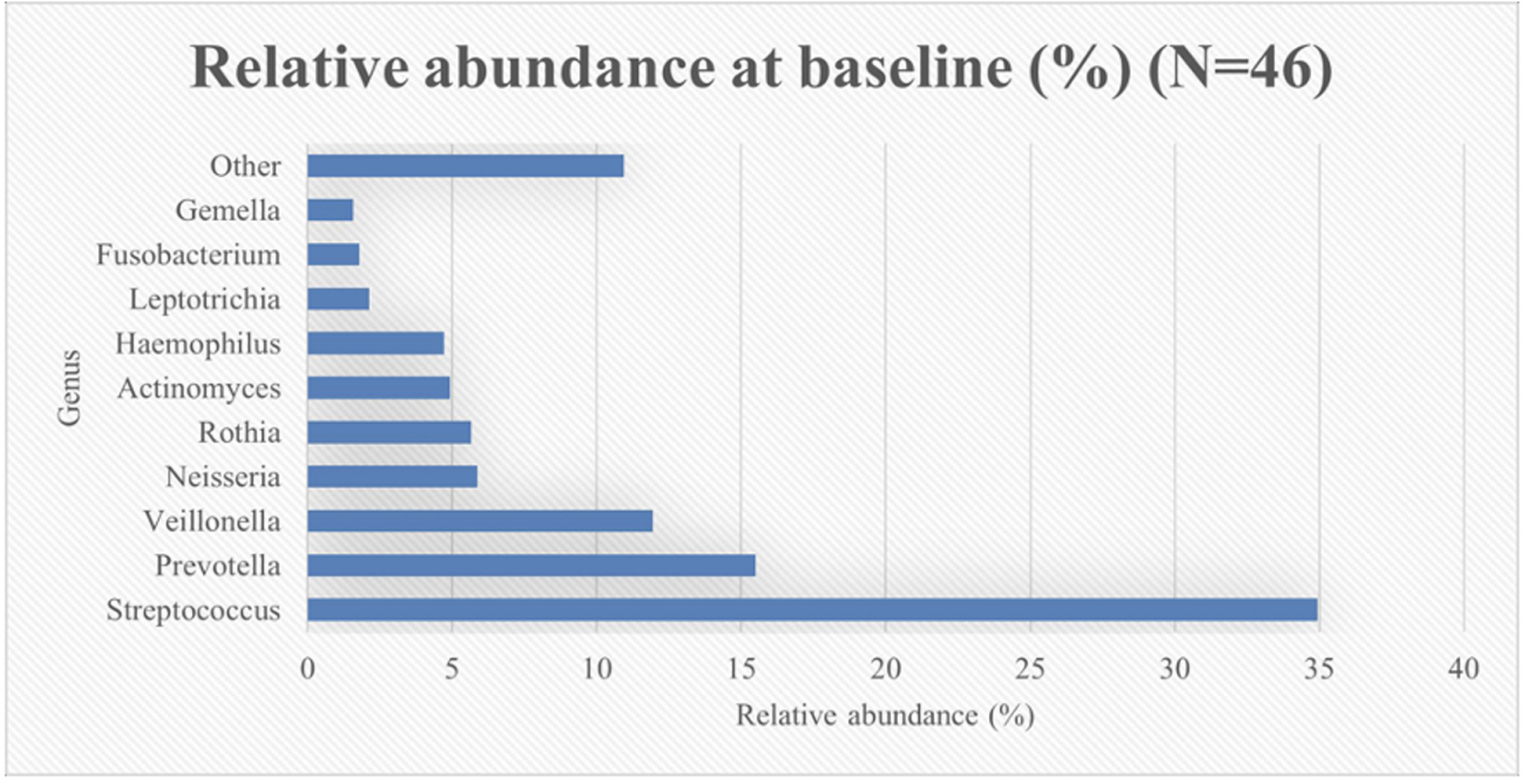
Relative genus abundance at baseline (%) (*N* = 46).

The top-10 most abundant genera at baseline were *Streptococcus, Prevotella, Veillonella, Neisseria, Rothia, Actinomyces, Haemophilus, Leptotrichia, Fusobacterium* and *Gemella* (Figure 2).

At baseline, the average (+ SD) Shannon index was 3.65 ± 0.46 (range 2.28–4.67). There was no difference in the average Shannon index by gender, CT regimen, patients with periodontal pockets ≤5 mm vs. pockets ≥6 mm, patients with or without a dental focus and different age groups (Mann Whitney *U*-test; Kruskal-Wallis test: *p* > 0.05). However, a significantly lower alpha-diversity was present in patients who never had smoked compared to those actively smoking or who quitted smoking (Kruskal-Wallis test, *p* = 0.04; quit vs. never *p* = 0.039, never vs. yes *p* = 0.050, quit vs. yes *p* = 0.704). No significant difference on the alpha-diversity of the oral microbiota was seen by the presence of an oral focus (linear mixed model, *p* = 0.144) or periodontal pockets ≥6 mm (linear mixed models, *p* = 0.720).

At baseline no significant difference was seen in the composition of the oral microbiota between gender, CT intensity and age groups (PERMANOVA, *p* > 0.05). However, there was a significant difference in microbial composition between three categories of smokers (PERMANOVA, *F* = 1.69, *p* = 0.0037), patients with vs. patients without an oral focus (PERMANOVA, *F* = 2.18, *p* = 0.0015), and patients with periodontal pockets ≤5 mm vs. ≥6 mm, (PERMANOVA, *F* = 2.02, *p* = 0.0039).

#### Oral microbiota before, during and after CT

**Figure 3:**
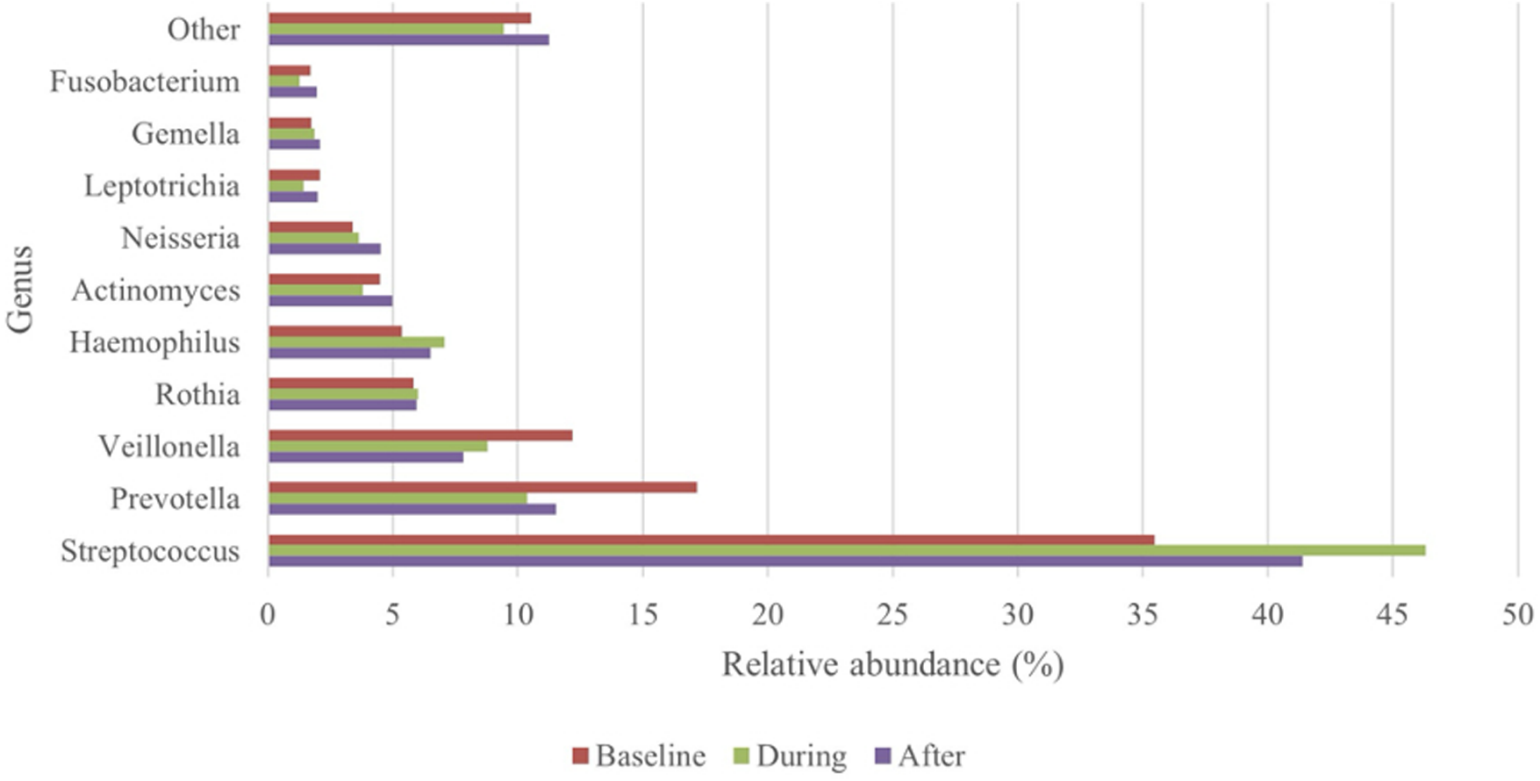
Top-10 most abundant genera: changes in relative abundance over the course of the treatment (*n* = 30).

For 30 patients oral rinsing samples were available that were collected before (baseline samples), during (CT-samples) and after CT (post-CT samples). During CT, oral rinsing samples were collected between days 8 to 73. The post-CT samples were collected between 40 and more than 200 days after the end of the CT regimen. Of the 30 patients included in this analysis, six patients developed OM grade 2 at some point during the course of CT treatment. At baseline, the top-10 most abundant genera (Figure 3) only slightly differed from the entire group at baseline (Figure 2). The prevalence in descending order was *Streptococcus, Prevotella, Veillonella, Rothia, Haemophilus, Actinomyces, Neisseria, Leptotrichia, Gemella* and *Fusobacterium*.

The average Shannon index was 3.67 ± 0.48 at baseline, 3.37 ± 0.60—during the treatment course and 3.59 ± 0.49—after the treatment was completed. Although the Shannon index was, on average, lower during CT, and recovered after CT, these changes were not significant (Wilcoxon Signed Rank test, *p* = 0.531).

The oral microbial composition differed significantly between baseline, during CT and after completion of the CT regimen (PERMANOVA using permutations restricted on Subject [*N* = 30, 90 samples, *F* = 0.95, *p* = 1e-4; post-hoc (no multiple-testing correction) for Phase 1vs 2, 1 vs. 3 and 2 vs. 3, respectively: *F* = 1.25, *p* = 0.0002; *F* = 0.90, *p* = 0.003; *F* = 0.71, *p* = 0.05].

To exclude the potential effect of OM in the changes of the oral microbiota, we analyzed the compositional changes and relative abundances without the 6 subjects who developed OM grade 2 over the course of the treatment. Again, a significant difference was seen (*N* = 24; *F* = 0.76; *p* = 0.0015; post-hoc for Phase 1 vs. 2, 1 vs. 3 and 2 vs. 3, respectively: *F* = 1.03, *p* = 0.0046; *F* = 0.77, *p* = 0.013; *F* = 0.49, *p* = 0.42).

**Figure 4:**
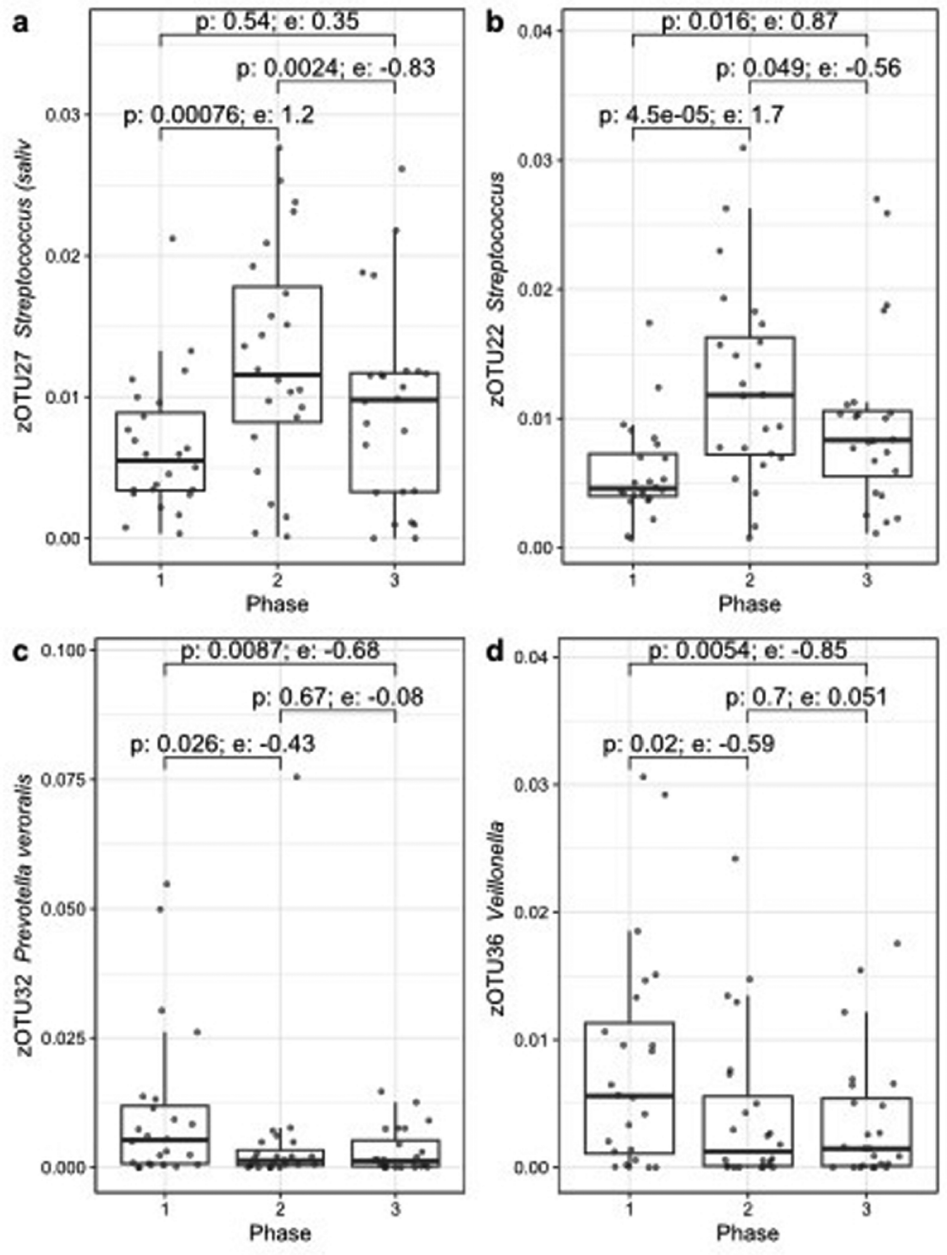
Four zOTUs showing significant changes in relative abundance before (phase 1), during (phase 2) or after CT (phase 3) over the course of the treatment, figures **(a–d)** are sorted by decreasing zOTU abundance. The *p*-value (Wilcoxon) and effect size (e) from ALDEx2 are indicated for each pair-wise comparison.

During the CT regimen, several zOTUs showed a significant difference in relative abundance by using ALDEx2, of which the four most abundant ones are shown in Figure 4. Only zOTU22 (*Streptococcus*) remained significant (Phase 1 vs. 2) after correction for multiple testing. However, the effect sizes of the shown zOTUs were large (>0.7 or <−0.7) for at least one pair-wise comparison.

#### Oral mucositis and oral microbiota

**Figure 5:**
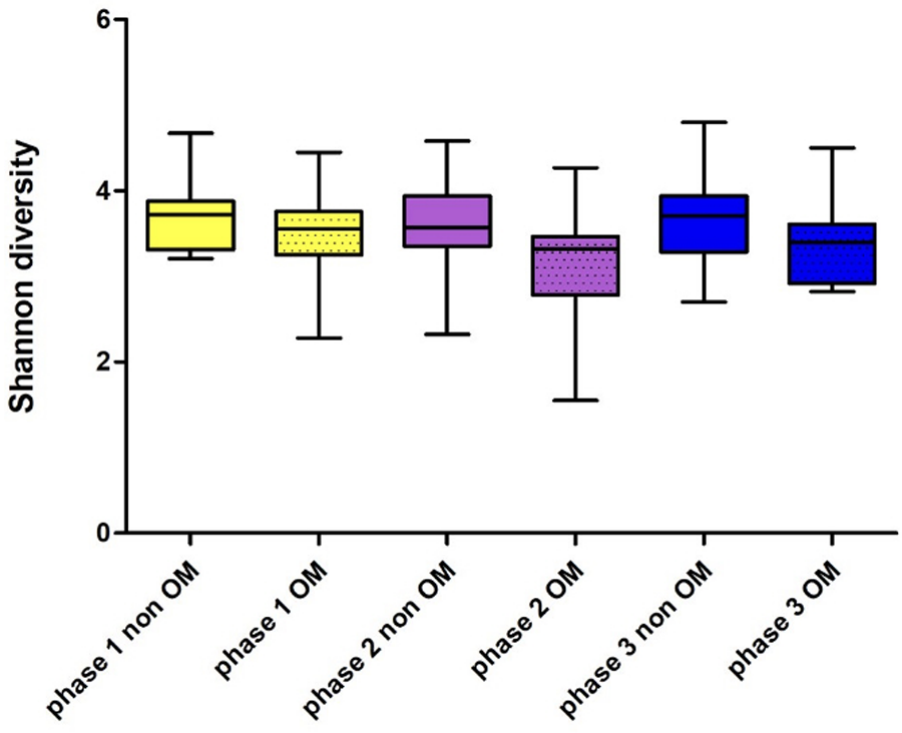
Shannon diversity of patients who did and did not develop oral mucositis before (phase 1), during (phase 2) and after CT (phase 3); OM: *n* = 15 (grade II), non OM: *n* = 30.

Of the 46 patients, 15 developed OM grade 2. The Shannon index of these 15 patients before the start of CT was 3.49 ± 0.49 and at the time point when they had developed ulcerative OM 3.19 ± 0.51 (Wilcoxon Signed Rank test, *p* = 0.083). When analyzing alpha-diversity data longitudinally, we found a significant linear effect of oral mucositis on the Shannon index (linear mixed model, 0.259124, *p* = 0.0001). Oral mucositis was associated with a lower Shannon index. Figure 5 shows the Shannon diversity of the patients who did not develop OM during the course of the treatment and patients who did develop OM grade 2.

There was a significant difference in the oral microbial composition in the 15 OM patients before the start of CT and at the moment they were diagnosed with OM [PERMANOVA 32767 restricted permutations (complete enumeration), *F* = 1.00, *p* = 0.0006].

**Figure 6:**
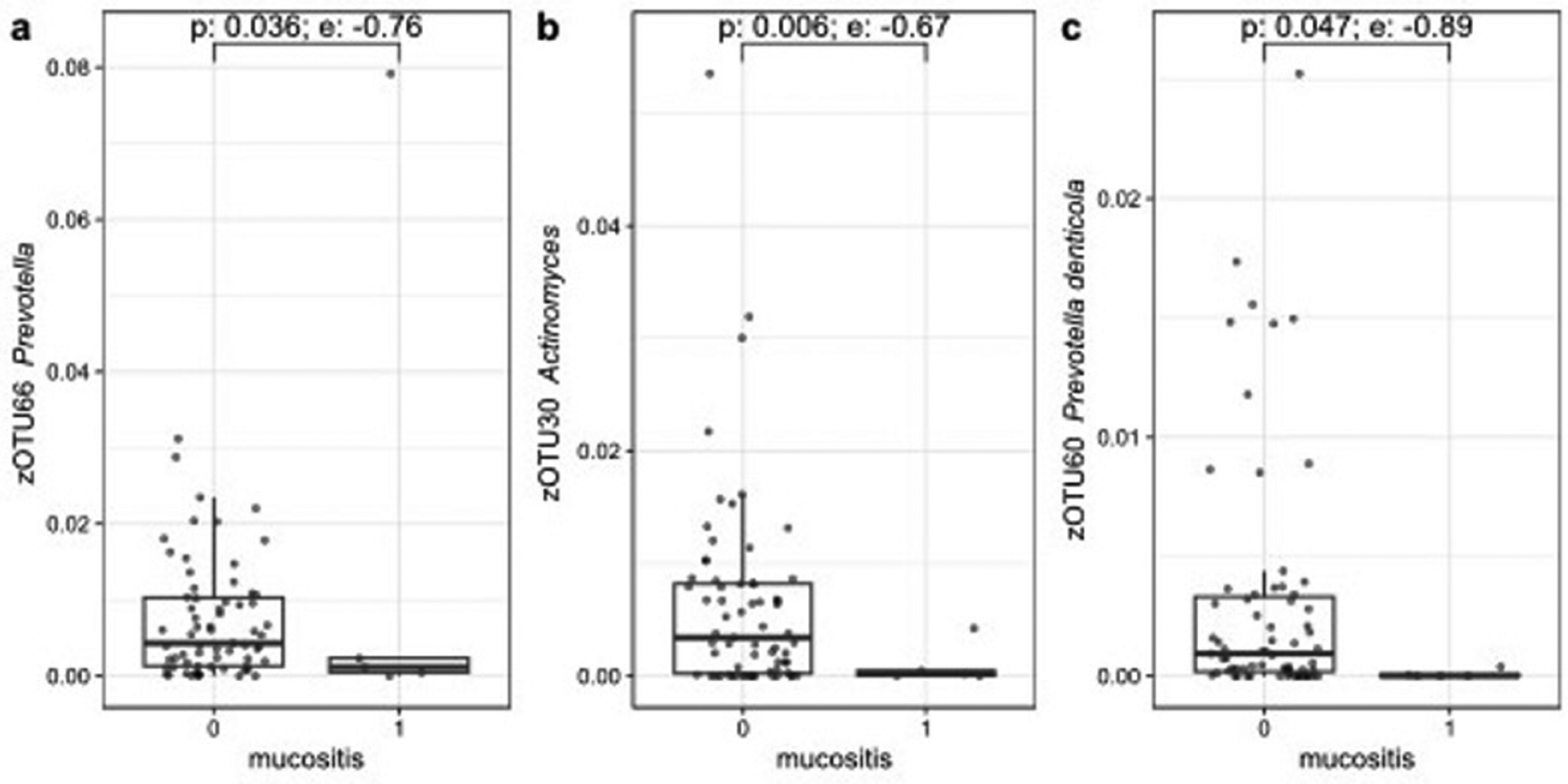
Three zOTUs showing significant changes in relative abundance between baseline and the moment of OM grade 2. Figures **(a–c)** are sorted on decreasing zOTU abundance. The *p*-value (Wilcoxon) and effect size (e) from ALDEx2 are indicated.

A decrease in relative abundance of eight zOTUs assigned to the genera of *Prevotella, Fusobacterium, Selenomonas, Actinomyces* and *Leptotrichia* was seen by using ALDEx2, during OM with an effect size less than −0.5. The three largest zOTUs, with effect sizes less than −0.7 are shown in Figure 6.

### Neutropenic fever and oral microbiota

Six out of the 46 patients developed neutropenic fever (13.0%). Two had an oral focus prior to the start of CT regimen. All but one developed OM grade 2 over the courses of the CT treatment. The Shannon diversity index was significantly lower at the moment of a fever (Wilcoxon Signed Rank test, *p* = 0.031; 3.55 ± 0.35 before the start of CT and 3.02 ± 0.64 at time of fever).

## Discussion

The aim of this study was to evaluate the oral microbial changes during myelosuppressive CT and possible associations with OM and FN in patients diagnosed with a solid tumor or lymphoma. We found that a lower alpha diversity of the oral microbiota and differences in beta diversity were significantly associated with OM. Additionally, a decrease was seen in the relative abundance of zOTUs assigned to the genera *Prevotella, Fusobacterium, Selenomonas, Actinomyces* and *Leptotrichia*, of which *Prevotella* and *Actinomyces* showed a greater effect size.

In general, the oral microbiota of our patient group at baseline was comparable with the general population as the abundance of the top 10 most abundant genera was similar with previously published findings (29, 50–52). A decrease in alpha diversity in relation to OM was also described by others (12, 21, 23, 53, 54). Hong et al. (12) concluded that shifts in the oral microbiota are strongly correlated with OM severity and Bruno et al. (53) found a significant difference in composition during OM.

At baseline, a significantly higher alpha diversity was seen in patients who smoked and a significant difference was seen in beta-diversity for smokers or patients with an oral focus including periodontitis. A higher alpha diversity is also reported in the review by Maki et al. (55) and the significant difference in beta diversity is also in line with the literature, as it is described that these factors can lead to dysbiosis of the oral microbiome (2, 20, 56).

During CT-regimen a decrease in alpha diversity was seen, but after completion of the treatment course a tendency to recover was also visible. However, these results were not significant. The decrease in alpha diversity is also reported by others (12, 21, 57–60). In contrast, Mougeot et al. (50) concluded in a review that CT treatment increases oral microbiome diversity and radiotherapy had the opposite effect. However, this review included different types of analysis for microbial identification, which potentially could lead to biased results. The tendency to recover after completion of the treatment is in line with Laheij et al. (23) who found a significant recovery of the oral microbiota after 3 months and stabilization of the oral microbiome after 1 year. Possibly, with a longer follow-up time, a significant recovery of the oral microbiota could also be demonstrated in our study.

Secondly, the beta diversity significantly differed over the course of the treatment. This effect remained after excluding patients who developed OM during the course of the treatment. However, no significant recovery in beta-diversity was seen after the course of the treatment was completed. Laheij et al. (23) did find a recovery of beta diversity to baseline levels after HCT, but they had a longer follow up time after the completion of cancer therapy. In contrast with our findings, Klymiuk et al. (29) found no changes over the course of the treatment in alpha and beta diversity, but clinical outcomes, like the development of OM, were not mentioned in the results. This could be a potential bias, as we and others, found a significant effect of OM in diversity changes (12, 57).

Significant changes were seen in relative abundance of zOTUs assigned to the genera of *Streptococcus*, *Veillonella* and *Prevotella*. *Streptococcus* and *Veillonella* are associated with oral health by preventing pathogenic bacteria to attach on the oral surfaces (61). *Prevotella* is an anaerobic commensal oral resident, but has been associated also with (extra)-oral disease (62). There is some evidence that suggesting that *Prevotella* may also have beneficial effects on human health, like for instance improved glucose control (63). Decrease of its relative abundance, as seen in the patients experiencing OM, may potentially reduce these benefits. *Actinomyces* is one of the genera that is predominantly present in the oral cavity and plays a crucial role in biofilm formation on teeth (64). However, the overall function of *Actinomyces* within the oral microbiome is not yet fully known. In a recent systematic review, Frey et al. (54) reported a decrease in relative abundance of *Prevotella* and *Actinomyces* during OM. So, our results conform to their findings.

Our study found a significantly lower alpha diversity in patients during a FN episode. To our knowledge, we are the first to study the oral microbiota in patients during a FN episode. The samples retrieved were all taken before or immediately after the start of antibiotic treatment for FN. However, these results are preliminary as only 6 patients could be included in this analysis. Furthermore, the potential role of the antibiotic treatment is not established. There is a lack of evidence on the role of the oral microbiome and the development of FN, as no research is published directly investigating microorganisms in blood and in the oral microbiome (20, 50). McMahon et al. (65) investigated the potential role of oral and gastrointestinal microbiome in bloodstream infections, however, they only analyzed the patients who had a positive blood culture. It is known that blood cultures of patients developing FN, are positive in only 0.2%–15% of cultures (66), so potential correlation could be missed. Moreover, several microorganisms present in the oral cavity cannot be cultured, or need different culture circumstances than are customary for regular blood cultures, such as longer incubation time or specific nutrients. For that reason, we obtained residual material from the blood collection during FN to analyze and identify potential oral microorganisms. However, the quality of the material was not sufficient, so no in-depth identification of microorganisms in blood could be performed. Furthermore, the blood cultures drawn during the FN episodes were all negative and no further analysis on cultured microorganisms could be performed. Sardzikova et al. (67) reported a significant relation of decreased alpha diversity in the gut microbiome in relation with FN. However, the studied patient group included paediatric patients undergoing hematopoietic stem cell transplantation, but these findings might give an indication of a potential relation of microbiome changes and the development of FN.

This study houses several limitations. The main limitation is the relatively small study population, due to the fact that the samples of 26 patient were not stored according to protocol, meaning that less robust conclusions can be drawn from our results. Furthermore, the patient group was heterogenic because of different tumor locations and different treatment regimens. However, this patient group encounters the majority of the cancer patients treated with myelosuppressive CT and no earlier study has been performed in this patient group. Only two studies (12, 29) investigated the oral microbiome changes in patients with solid tumors, of which one (29) only included breast cancer patients and the other study selected on the type of CT, not mentioning tumor diagnosis.

Another limitation of this study is the absence of a (healthy) control group. Therefore, we could not establish if the tumor itself had an influence on the oral microbiota. But the baseline composition of the oral microbiome showed no large deviation to what is known in healthy individuals. Even with these limitations, this study brought results about the oral microbiome of a specific patient group, where data is scarce. Further larger longitudinal studies are needed to draw more robust conclusions about the possible relation between febrile neutropenia, where the duration and depth of the neutropenic episodes also needs to be further analyzed; oral mucositis and the oral microbiota.

In conclusion, in patients treated with myelosuppressive chemotherapy for solid tumors, a lower alpha diversity and changed beta diversity of the oral microbiota was significantly associated with oral mucositis. Furthermore, a change of the oral microbiota occurs during episodes of febrile neutropenia. However, these results should be interpreted with caution because of low number of participants. Further study is needed to draw more robust conclusions and to identify bacteria (that are often present in the oral cavity) in blood during febrile neutropenia.

## Data Availability

The raw data supporting the conclusions of this article will be made available by the authors, without undue reservation.

## Appendix 1

**Table:**

Tumor subgroup	CT regimen	Relatively high/low risk	No of patients
Gynecological	Carboplatin AUC5, liposomal doxorubicin 30 mg/m^2^	Low	1
	Carboplatin AUC 4, Gemcitabin 1,000 mg/m^2^	Low	1
	Carboplatin AUC 6, Paclitaxel 175 mg/m^2^	Low	2
	Cisplatin 40 mg/m^2^	Low	13
	Bevacizumab 15 mg/kg, Cisplatin 50 mg/m^2^, Paclitaxel 175 mg/m^2^	Low	1
	Cisplatin 80 mg/m^2^, Etoposide 100 mg/m^2^	High	1
	Liposomal Doxorubicin 45 mg/m^2^	Low	1
Upper GI	Carboplatin AUC 2, Paclitaxel 50 mg/m^2^	Low	3
	Carboplatin + Paclitaxel + Trastuzumab + Pertuzumab	Low	1
	Cisplatin 80 mg/m^2^, Etoposide 100 mg/m^2^	High	1
	Capecitabin 1,000 mg/m^2^, Epirubicin 50 mg/m^2^, Oxaliplatin 130 mg/m^2^	High	1
	5FU 400 mg/m^2^ + continuously 2,400 mg/m^2^ for 46 h, Oxaliplatin 85 mg/m^2^, Irinotecan 180 mg/m^2^, Leucovorin 400 mg/m^2^	High	2
	Gemcitabin 1,000 mg/m^2^, NAB-Paclitaxel 125 mg/m^2^	Low	1
Sarcoma	Cisplatin 60 mg/m^2^, Doxorubicin 37, 5 mg/m^2^, MTX 12 g/m^2^	High	2
	Dactinomycin 2 mg, Ifosfamide 3 gr/m^2^, Vincristine 2 mg Doxorubicin 30 mg/m^2^	High	1
	Dactinomycin 2 mg, Ifosfamide 3 gr/m^2^, Vincristine 2 mg	High	2
Lymphoma	Doxorubicin 20 mg/m^2^, Etoposide 150 mg/m^2^, Ifosfamide 3,000 mg/m^2^, Vincristin 2 mg	High	2
	Bleomycin 10 USP, Dacarbazin 375 mg/m^2^, Doxorubicin 25 mg/m^2^, Vinblastin 6 mg/m^2^	High	1
	DA-EPOCH-R (Cyclophosphamide Etoposide, Prednisolon, Vincristin, Hydroxoanurubicine, Rituximab) + MTX it	High	1
	Cyclophosphamide 750 mg/m^2^, Doxorubicin 50 mg/m^2^, MTX high dose, Rituximab 375 mg/m^2^, Vincristin 2 mg	High	1
	Cyclophosphamide 750 mg/m^2^, Doxorubicin 50 mg/m^2^, Prednison, Rituximab 375 mg/m^2^, Vincristine 2 mg	High	1
Urinary	Bleomycin 30USP, Cisplatin 20 mg/m^2^, Etoposide 100 mg/m^2^	High	2
	Carbazitaxel 25 mg/m^2^, Prednison 5 mg 2dd	Low	1
Breast	Cyclophosphamide 600 mg/m^2^, Doxorubicin 60 mg/m^2^, Paclitaxel 80 mg/m^2^	High	2
Lower GI	Folfiri: 5FU + Irinotectan: Irinotecan 180 mg/m^2^, Folineacid 400 mg/m^2^, Fluorouracil 400 mg/m^2^	High	1
			46

5FU, 5-Fluoruracil; USP, United States Pharmacopeia; MTX, methotrexate; AUC, area under the curve; NAB, nanoparticle albumin-bound; It, intrathecal.
